# Development of a Custom MALDI-TOF MS Database for Species-Level Identification of Bacterial Isolates Collected From Spacecraft and Associated Surfaces

**DOI:** 10.3389/fmicb.2018.00780

**Published:** 2018-05-18

**Authors:** Arman Seuylemezian, Heidi S. Aronson, James Tan, Mandy Lin, Wayne Schubert, Parag Vaishampayan

**Affiliations:** Biotechnology and Planetary Protection Group, Jet Propulsion Laboratory, California Institute of Technology, Pasadena, CA, United States

**Keywords:** MALDI-TOF MS, bacterial phylogenetics, space microbiology, cleanroom, taxonomy

## Abstract

Since the 1970s, the Planetary Protection Group at the Jet Propulsion Laboratory (JPL) has maintained an archive of spacecraft-associated bacterial isolates. Identification of these isolates was routinely performed by sequencing the 16S rRNA gene. Although this technique is an industry standard, it is time consuming and has poor resolving power for some closely related taxa. Matrix-assisted laser desorption/ionization time of flight (MALDI-TOF) mass spectrometry is widely used in clinical diagnostics and is a promising method to substitute standard 16S rRNA sequencing. However, manufacturer-provided databases lack the bacterial diversity found in spacecraft-assembly cleanrooms. This study reports the development of the first custom database of MALDI-TOF MS profiles of bacterial isolates obtained from spacecraft and associated cleanroom environments. With the use of this in-house database, 454 bacterial isolates were successfully identified in concurrence with their 16S rRNA sequence-based classifications. Additionally, MALDI-TOF MS resolved strain-level variations, identified potential novel species and distinguished between members of taxonomic groups, which is not possible using conventional 16S rRNA sequencing. MALDI-TOF MS has proved to be an accurate, high-throughput approach for real-time identification of bacterial isolates during the spacecraft assembly process.

## Introduction

The Committee on Space Research maintains the planetary protection policy for the protection of Earth and other planets from biological contamination. It is important to preserve planetary conditions for future exploration by preventing terrestrial microorganisms from contaminating other planetary bodies, including Mars and Europa, which are of particular interest for life-detection experiments. When assembling spacecraft, microbial bioburden requirements must be met to minimize forward contamination. The NASA Standard Assay (NSA) is the method employed to continually monitor the microbial bioburden present on spacecraft hardware and associated surfaces throughout the assembly, testing, and launch operations of the flight project. Surface samples obtained from spacecraft and spacecraft-assembly cleanrooms are heat-shocked, and bacterial survivors of this assay are used as a proxy for total microbial bioburden. Survivors of the NSA are often more resistant to sterilization techniques, making them more useful in understanding the potential of microbes to survive in the extreme conditions of space. Planetary protection engineers archive these isolated microorganisms for long-term storage to be used in research studies that may better assess forward contamination concerns.

The Biotechnology and Planetary Protection group at NASA’s JPL has archived over 5,000 bacterial isolates from missions dating as far back as the Viking 1 mission in 1975. Proper taxonomic characterization of spacecraft-associated microbes is crucial to understanding their metabolic capabilities and survivability under various conditions. Ultimately, understanding the microbial communities on spacecraft surfaces will allow researchers to better minimize the probability of forward contamination by improving sterilization procedures (Moissl et al., 2007).

Bacterial isolates in the Planetary Protection (PP) archive are systematically identified using 16S rRNA gene sequencing. Although 16S rRNA sequence-based bacterial identification is the current industry standard, it is time consuming and has low phylogenetic resolution at the species level and poor discriminatory power for some genera (Case et al., 2007; Janda and Abbott, 2007). An alternative approach to microbial characterization that is currently increasing in use is matrix-assisted laser desorption/ionization time-of-flight (MALDI-TOF) mass spectrometry (MS) (Bizzini et al., 2011). MALDI-TOF MS produces mass spectral peaks by generating ions from proteins, peptides, and oligonucleotides without fragmentation, allowing them to travel through a flight tube within a specific time based on their mass to charge ratio (Vargha et al., 2006). These protein profiles, which are primarily based on ribosomal proteins, can be used for microbial identification by generating a database of mass spectral profiles from known bacterial species (Ryzhov and Fenselau, 2001). MALDI-TOF MS has numerous benefits over 16S rRNA sequencing: it is rapid, high-throughput, and has a much higher resolving capability than 16S rRNA sequencing (Mellmann et al., 2008; van Veen et al., 2010; Bizzini et al., 2011).

The use of MALDI-TOF MS is currently expanding in clinical laboratories due to its rapid and accurate bacterial identification capabilities. In previous studies, it was shown that bacterial identification using MALDI-TOF MS was significantly better than conventional biochemical systems for correct species identification (Seng et al., 2009; van Veen et al., 2010; Bessede et al., 2011; Bizzini et al., 2011). Any discrepancies that were observed were associated with an absence of sufficient reference spectra in the MALDI-TOF MS database, and it was concluded that MALDI-TOF MS can be implemented easily for routine identification of bacteria in a medical microbiology laboratory (Carbonnelle et al., 2012; Rahi et al., 2016). The slow progression in use of this technique for bacterial identification in other industries is due to the limited availability of relevant mass spectral libraries for use, and the bias of existing libraries towards clinical isolates (Vargha et al., 2006).

The initial identification of isolates from the JPL PP archive using the manufacturer-provided database (Bruker Corp.) yielded successful identification of only 8% of bacterial isolates in accordance with their 16S rRNA characterizations (data not shown). Because of rigorous microbial reduction techniques and the selective nature of the NSA, the diversity of microbial communities found on spacecraft and associated surfaces is extremely limited and mainly contains spore-forming bacterial species belonging to the *Bacillus* genus. This genus is not well-represented within the manufacturer-provided MALDI-TOF database.

This technical bottleneck was overcome by creating a custom in-house database of MALDI-TOF main spectral profiles (MSPs) using a reproducible standard operating procedure. This database is comprehensive and representative of isolates present in the JPL PP archive, and is the first to contain isolates from the world’s largest collection of spacecraft-associated microorganisms. Implementation of this custom MALDI-TOF MS database will allow for a much more efficient, rapid, accurate, sensitive, and less-redundant bacterial archiving workflow. In this study, we claim that MALDI-TOF was able to resolve the differences among taxa that cannot be differentiated solely by 16S rRNA sequences. We have also shown the increased resolution of MALDI-TOF MS in revealing strain level differences among isolates deemed identical by 16S rRNA sequencing.

## Materials and Methods

### Bacterial Isolates

Samples were collected from flight hardware and associated surfaces from spacecraft-assembly cleanrooms during various NASA missions using Texwipe TX3211 wipes and Puritan 806-WC cotton swabs. Samples were heat shocked at 80°C for 15 minutes, pour plated on TSA plates, incubated at 32°C for 72 hours, and sub-cultured to obtain pure cultures (National Aeronautics and Space Administration, 2010).

### 16S rRNA Sequencing

Colony PCR was performed on 454 bacterial isolates. 16S rRNA gene was amplified using 27F (5′-AGA GTT TGA TCC TGG CTC AG-3′) and 1492R (5′-GGT TAC CTT GTT ACG ACT T-3′) primers using PCR under the following conditions: 95°C for 5 min; 34 cycles of 95°C for 50 s, 55°C for 50 s, 72°C for 55 s; then 72°C for 10 min (Frank et al., 2008). To remove excess dNTPs and primers from amplified PCR products, 20 units Exonuclease I (New England Biolabs, Ipswich, MA, United States), 5 units Antarctic Phosphatase (New England Biolabs, Ipswich, MA, United States), and 10 μl ddH2O were added to 10 μl amplified PCR product. PCR products and enzyme mix were incubated at 37°C for 45 min followed by 85°C for 15 min to inactivate the enzyme. PCR products were sequenced bi-directionally to obtain near full length 16S rRNA sequences (∼1,400 bp) using Sanger sequencing at Macrogen Corporation (Rockville, MD, United States) and sequence data were processed using Lasergene software (DNASTAR, Wisconsin, United States).

### Taxonomic Classification

The phylogenetic classification of all bacterial isolates included in this study was confirmed using the SILVA LTP type strain database, a quality-controlled database of prokaryotic 16S rRNA sequences from type strains (Quast et al., 2013; Yilmaz et al., 2014; Glockner et al., 2017). Sequences with ≥98.7% sequence identity to a database entry were identified at the species level. Sequences with >95% sequence identity to a database entry were identified at the genus level (Schloss et al., 2009; Kim et al., 2014; Parulekar et al., 2017; Tourlousse et al., 2017).

### Selection of Representatives Bacterial Isolates

Sequence orientation was checked by aligning sequences in reference to the SILVA ribosomal RNA database release 123 using the align.seqs command in mothur (Schloss et al., 2009). The reverse complement of a sequence was used if sequence similarity in reference to the database was less than 50% in the original orientation. A distance matrix was produced using dist.seqs and sequences were clustered at 99% similarity using the cluster command with a neighbor joining algorithm. A representative for each cluster (OTU) was chosen using the get.oturep command. Eighty four unique OTUs were generated.

### MALDI-TOF MS Sample Preparation

Isolates were revived from cryobead or glycerol-preserved stocks from the PP archive and grown on TSA plates for ∼24 hours or until visible growth was observed. To avoid sporulation, MALDI-TOF MS was performed immediately after the growth was observed on the TSA plate. Isolates were spotted from a single colony onto MALDI-TOF MS 48-well target plates using a sterile toothpick following Bruker specifications. One microliter of 70% formic acid was layered on top of the sample and air-dried. One microliter of α-Cyano-4-hydroxycinnamic acid matrix in 50% acetonitrile–2.5% trifluoroacetic acid was added to each spot and air-dried.

### Creation of Main Spectral Profiles (MSPs) Using MALDI-TOF

An MSP database entry was created for one representative isolate from each of the 84 unique OTUs present in the current study, as well as 47 MSPs from type species isolates procured from Deutsche Sammlung von Mikroorganismen und Zellkulturen (DSMZ, Germany) and Agricultural Research Service Culture Collection (NRRL, United States) microbial culture collections. MSPs were created following manufacturer’s suggestions, but at higher levels of stringency. Isolates for MSP creation received eight biological replicates on the target plate and were prepared as previously described, and spectra were obtained in three separate intervals per target spot (Zhang et al., 2015). MALDI-TOF MS was performed using a microflex LT bench-top mass spectrometry instrument (Bruker Daltonics, Billerica, MA, United States). Processing of raw spectral data, including baseline subtraction, smoothing, and outlier and flat line elimination was performed using flexAnalysis software (Bruker Daltonics, Billerica, MA, United States). MALDI BioTyper software was used to calculate a main spectrum using at least 10 spectra for each isolate (a minimum of five spectra is recommended by Bruker). Each spectral line that constituted the MSP was verified to have a log score greater than 2.7 and a peak frequency greater than 75%.

### Real-Time Classification (RTC) of Bacterial Isolates

A total of 454 isolates were identified using MALDI-TOF MS and the Bruker Real Time Classification software. Isolates were prepared using a direct transfer technique and were identified in four biological replicates. The Bruker Real Time Classification software was used to compare spectra generated from each isolate to both the PP custom database and the manufacturer provided BDAL spectral library containing a total of 7,281 MSPs.

### Scoring of Validation Experiment

Real Time Classification results were analyzed based on the log scores they received. Log scores ≥2.2 consistently across a minimum of three replicates were considered to be a reliable species level identification. Scores of <2.2 were considered as unidentified/potentially novel species.

### Statistical Analyses

Maximum likelihood phylogenetic trees of 16S rRNA sequences were constructed using MEGA 7.0.20 (Center for Evolutionary Medicine and Informatics, Tempe, AZ, United States) with the lowest BIC (Bayesian Information Criterion) score-containing model. Composite Correlation Index (CCI) and MSP dendrograms were constructed using the MALDI Biotyper Compass Explorer 4.1. Build 60. At least two spectra were used to represent each strain’s mass spectral profile (2.7 log score). CCI parameter intervals were set at 10, and the mass lower bound and upper bound were 3,000 and 12,000 kDa, respectively. Dendrograms were constructed using a Euclidean distance measure and an average linkage algorithm. 16S rRNA sequences of type strains were acquired through the Silva LTP Type Strain Database.

## Results

A total of 454 16S rRNA sequences were binned into 84 operational taxonomic units at 99% sequence similarity using mothur (Schloss et al., 2009) belonging to a total of 51 distinct species and 14 distinct genera. At the genus level, 85% of these sequences were identified as *Bacillus*, 3.3% were *Brevibacterium*, and 3.8% were *Staphylococcus*, with the remaining 7.9% belonging to 11 other genera. An MSP was generated for a single representative isolate from all 84 OTUs, and validated using real-time classification.

**FIGURE 1:**
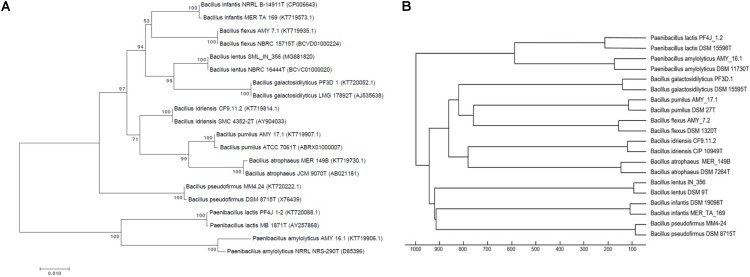
Neighbor-joining tree based on nearly complete 16S rRNA gene sequences **(A)** and Hierarchical clustering using Euclidean distance and average linkage algorithm of mass spectra **(B)** of 10 isolates from the PP archive and corresponding type strains. Mass spectra of PP isolates cluster with corresponding type strain MSPs, in concurrence with 16S rRNA.

In order to demonstrate the concurrence between phylogenies generated by both 16S rRNA and MALDI-TOF MS data, a neighbor-joining phylogenetic tree was constructed using nearly-complete 16S rRNA sequences of 10 isolates from this study and their corresponding type-strains (**Figure 1A**). A hierarchical dendrogram was also generated using MSPs of these isolates and their type-strain MSPs from the Bruker database (**Figure 1B**). Mass spectra of these isolates clustered with their corresponding type-strain MSPs. The tree constructed using 16S rRNA sequences (**Figure 1A**) showed the same overall clustering that was observed in **Figure 1B**, which provides higher confidence for MALDI TOF MS-based identifications. Although the overall formation of clades is equivalent, the tree topology is not identical. These differences are due to differences in algorithms used to construct the phylogenetic tree and dendrogram. While 16S rRNA sequencing analyzes only a single ∼1,500 bp gene, MALDI-TOF MS analyzes a larger spectrum of protein peptides present in a bacterial cell. This allows for the detection of proteomic differences across bacterial cells not considered by 16S rRNA sequencing.

### Resolution of Strain-Level Variation

**FIGURE 2:**
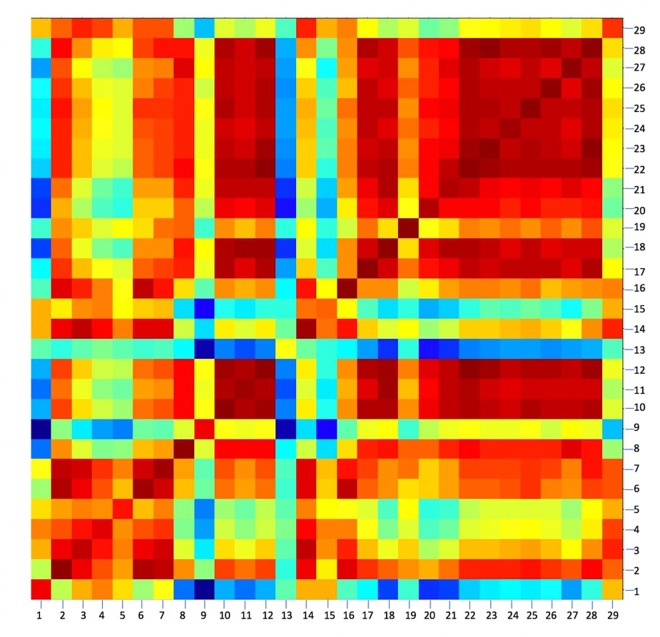
A composite correlation index (CCI) of 29 bacterial strains identified as *Bacillus firmus* (≥98.7% 16S rRNA sequence similarity). Strain-level variations were seen using CCI clustering, although they are 100% similar based on 16S rRNA sequences. At least two spectra were used to represent each strain’s mass spectral profile (≥2.7 log score). MALDI-TOF Biotyper Compass Explorer V4.1, Euclidean distance, and 10 intervals were used. Distances between mass spectra are represented as a color spectrum ranging from red, high similarity, to blue, low similarity.

Because MALDI-TOF MS captures a larger protein profile than what is represented by the 16S rRNA gene, it can resolve strain-level differences between bacterial isolates. This was demonstrated using the most highly-represented species present in this study, *Bacillus firmus*. A total of 29 representative strains of *B. firmus* belonging to seven different OTUs were used to construct a CCI (**Figure 2**). Scores of >0.9, shown in red, indicate a strong correlation between the MALDI-TOF MS spectra of two isolates (Bruker, Massachusetts, United States). Scores of 0, shown in blue, indicate no correlation between isolates. CCI analysis using MALDI-TOF spectral data showed strain-level variation between isolates that were initially identified as 100% identical by 16S rRNA sequences.

### Resolution of Taxonomic Groups

Certain taxonomic groups are indistinguishable by 16S rRNA sequencing because their 16S rRNA sequences are nearly identical (>99% sequence similarity; Bosshard et al., 2006; Mignard and Flandrois, 2006; Janda and Abbott, 2007). All members of the *Bacillus pumilus* taxonomic group, which is comprised of *B. pumilus*, *B. safensis*, *B. australimaris*, and *B. zhangzhouensis* (99.9% 16S rRNA gene sequence similarity) are present in the PP archive (Satomi et al., 2006). The taxonomic identifications of 10 bacterial isolates from the *B. pumilus* group based on 16S rRNA sequencing and MALDI-TOF were compared (**Table 1**). Four of the ten isolates (PF9-10.2.1, PF9-10.1.1, MER_TA_110.2, and AMY_31.2) had 16S rRNA identifications that were in agreement with their MALDI-TOF MS-based identifications, confirmed by log scores >2.2. Three isolates (AMY_17.1, MER_178, and MER_114.2) had RTC scores >2.2 that were not in concurrence with their 16S rRNA identifications, and can confidently be classified by their MALDI-TOF MS-based identifications using the MSPs prepared from type strains. In addition to providing increased resolution of taxonomic groups, MALDI-TOF MS may be useful in the detection of novel species. Three isolates (MSL_3001, IN_293, and V45.5) were considered potentially novel species within the *B. pumilus* taxonomic group due to log scores being <2.2.

**Table 1:** Comparative taxonomic identification of 10 bacterial isolates belonging to the *Bacillus pumilus* taxonomic group (comprised of *B. pumilus, B. safensis, B. zhangzhouensis*, and *B. australimaris*). MALDI-TOF MS analysis was able to resolve taxonomic identifications of isolates that are indistinguishable by 16S rRNA sequences.

Isolate name	16S rRNA identification	Percent similarity	MALDI-TOF RTC identification	Log score similarity
PF9-10.2.1 (KT920027.1)	*B. safensis*	100	Bacillus safensis_FO36b^T^	2.3
PF9-10.1.1 (KT720019.1)	*B. safensis*	100	Bacillus safensis_FO36b^T^	2.22
MER_TA_110.2 (KT719518.1)	*B. safensis*	99.4	Bacillus safensis_FO36b^T^	2.32
AMY_31.2 (KT719925.1)	*B. pumilus*	99.2	Bacillus pumilus_DSM 27^T^	2.33
AMY_17.1^∗^ (KT719907.1)	*B. pumilus*	99.8	Bacillus australimaris_LMG_27697^T^	2.32
MER_178^∗^ (KT719762.1)	*B. australimaris*	99.8	Bacillus safensis_FO36b^T^	2.24
MER_114.2^∗^ (KT719690.1)	*B. zhangzhouensis*	99.3	Bacillus pumilus_DSM 27^T^	2.36
MSL_3001^∗†^ (KT719852.1)	*B. pumilus*	99.8	Bacillus australimaris_LMG_27697^T^	2.19
IN_293^∗†^ (MG881821)	*B. australimaris*	99.8	Bacillus safensis_FO36b^T^	2.17
V45.5**†** (KT720340.1)	*B. pumilus*	99.8	Bacillus pumilus_DSM 27^T^	2.03

*^∗^Mismatch between 16S rRNA and MALDI-TOF identifications. ^†^Potential novel species indicated by a log score <2.2. ^T^Type strain.*

### 16S rRNA Concurrence With MALDI-TOF MS-Based Identification

Of the 454 microbial isolates identified using both 16S rRNA sequencing and MALDI-TOF MS based bacterial identification, 454 isolates were identified to the genus level using 16S rRNA sequencing (>95% sequence similarity), and 420 of which were identified to the species level (>98.7% sequence similarity with closest type species). Both identification methods indicate 34 strains belonging to potentially novel species.

All 454 isolates identified at the genus level by 16S rRNA sequencing agree with MALDI-TOF MS based genus level identification. Of the 420 isolates identified to the species level with 16S rRNA sequencing, 244 concur with their MALDI-TOF MS-based identification. Though the other 75 isolates agree at the genus level, MALDI-TOF MS identification suggests that they belong to potentially novel microbial species. The remaining 101 isolates belong to taxonomic groups and were identified to closely related microbial species within the same genera based on 16S rRNA sequencing and were difficult to resolve taxonomically in the absence of type strain spectra.

## Discussion

The Biotechnology and Planetary Protection group at NASA’S JPL works to ensure that biological cleanliness requirements are met on spacecraft. Bioburden levels on spacecraft surfaces are assessed using the NSA, and survivors from this assay are identified and archived. In the past, 16S rRNA-based bacterial identification was implemented to identify archived microbial isolates. Though it is a gold standard, 16S rRNA sequencing has several intrinsic disadvantages, including time and inadequate resolution of closely related microbial taxa.

MALDI-TOF MS has been implemented in diagnostic microbiology laboratories as a rapid, high-throughput bacterial identification technique (Bizzini et al., 2011). The success of this identification technique, however, is dependent on the reference strains found in the mass spectral database (van Veen et al., 2010; Rahi et al., 2016). Due to the clinical bias of available MALDI-TOF MS databases, it has been necessary for other researchers to establish custom reference databases of bacterial strains relevant to their studies (Mellmann et al., 2008; Bizzini et al., 2011; Calderaro et al., 2014; Rahi et al., 2016). In studies implementing MALDI-TOF MS-based bacterial identification, 16S rRNA identification was used as a reference technique to validate the accuracy of the custom databases (Bizzini et al., 2011; Calderaro et al., 2014). Since most of these other databases are inaccessible, proprietary and often costly to access, little spectral information is shared among researchers. In this study, we report the first ever and largest custom MALDI-TOF MS database of bacterial isolates from spacecraft and associated surfaces.

### Resolution of Strain Variation

It has been widely established in the literature that characterizing a microorganism based on 16S rRNA sequencing requires ≥98.7% sequence similarity for species-level identification, and ≥95% sequence similarity for genus-level identification (Nguyen et al., 2016). In previous studies, MALDI-TOF MS based identifications yielded discordant results with those of 16S rRNA; however further phenotypic methods of identification were in agreement with MALDI-TOF MS based identification, further elucidating the limited resolution of 16S rRNA sequences (Bizzini et al., 2011). MALDI-TOF MS has a higher resolving capability than that of 16S rRNA sequencing as it analyzes a wider range of proteins than just the sequence of the 16S ribosomal subunit. MALDI-TOF MS has been applied in clinical studies to rapidly identify pathogenic strains of bacteria (Dieckmann and Malorny, 2011). In environmental microbial ecology studies, MALDI-TOF MS could be used to characterize isolates with much higher accuracy than that of 16S rRNA sequencing.

In order to capture strain-level variations among isolates within the PP archive and test the resolution of MALDI-TOF MS, 84 OTUs were defined at 99% sequence similarity. From the 454 isolates used in this study, 18% were identified by 16S rRNA sequencing as *B. firmus*. CCI analysis of 29 isolates with 100% identical 16S rRNA sequences shows distinct differences between strains (**Figure 2**). The ability to accurately characterize strain-level differences is useful in understanding differences across redundantly isolated strains identified as the same species across multiple missions, such as those isolated from spacecraft hardware and associated surfaces. MALDI-TOF MS may be more useful than conventional 16S rRNA sequencing for identifying and grouping isolates based on strain level variations.

### Resolution of Taxonomic Groups

Although 16S rRNA gene sequencing is the gold standard of bacterial identification, it has poor resolving power at the species level due to its limited genomic representation. Some bacterial species yield ambiguous taxonomic identifications using 16S sequencing due to their high sequence similarity to other species. These species are referred to as taxonomic groups, and cannot be differentiated solely by their 16S rRNA sequences (Stackebrandt and Goebel, 1994; Venkateswaran et al., 1999; Satomi et al., 2006). For example, *Bacillus safensis, B. pumilus, Bacillus zhangzhouensis*, and *Bacillus australimaris* all share >99.9% sequence similarity in their 16S rRNA sequences, but their entire genomic DNA sequences share only 54–66% homology (Satomi et al., 2006; Liu et al., 2015). In other cases, such as the species *Aeromonas veronii*, the bacterial genome can contain up to six different copies of the 16S gene that all differ by up to 1.5% (Janda and Abbott, 2007). This can yield different identifications depending on which copy was amplified, thus making 16S rRNA sequence identification ambiguous and inaccurate. This limited resolution and uncertainty in 16S rRNA-based bacterial identification has been resolved by sequencing other house-keeping genes such as *rpoB* or *gyrB*, which can have higher phylogenetic resolution than the 16S rRNA gene (Case et al., 2007; Persson et al., 2015). However, these techniques still employ the lengthy, labor-intensive processes of cultivation and isolation, cell harvesting, DNA extraction, PCR, gel electrophoresis, amplicon purification, sequencing, and sequence analysis.

While 16S rRNA sequence identification is based only on a gene of 1,500 or fewer base-pairs, MALDI-TOF MS captures unique molecular signatures that are representative of a larger range of proteins and can clearly distinguish differences between two closely related species. Over 20% of the microbial isolates from this study belong to taxonomic groups, and the ability to resolve the identity of these ambiguous species using mass spectra will allow for more accurate identifications. The 47 type strains from bacterial taxa present in this study were procured from various culture collections (DSMZ and NRRL) and used to make MSPs. Since type strains are thoroughly characterized using multiple techniques (i.e DNA-DNA hybridization, 16S rRNA sequencing, biochemical analyses, cell wall analysis, and fatty acid analyses), there is increased confidence in their taxonomic affiliation. By including MSPs from these type species strains in our database, we have increased confidence in MALDI-TOF MS based identification.

The *B. pumilus* taxonomic group that includes *B. safensis, B. pumilus, B. australimaris*, and *B. zhangzhouensis* was used as a case study (Liu et al., 2015). Taxonomic identifications based on both 16S rRNA sequences and MALDI-TOF MS were compared (**Table 1**). Isolates that had concurrent identifications using these methods (with RTC log scores >2.2) were considered to be accurately identified. Isolates that did not have matching identifications with each method, but had log scores >2.2 were considered to be accurately identified by their MALDI-TOF MS based classification. Any isolate that was identified to members of this taxonomic group based on 16S rRNA sequences, but had MALDI-TOF MS based log scores <2.2 were considered to be potentially novel species. Strains that are potentially novel species and novel taxonomic group members will be falsely identified using 16S rRNA gene sequencing because they will often match to other closely related species with high sequence similarities. However, these potentially novel species will receive low MALDI-TOF scores (<2.2), even with the presence of MSPs from other closely related species, allowing for accurate detection of novel species. Storing and re-analyzing spectra using Bruker software will facilitate periodic screening of previously unidentified strains against an updated database.

In addition to providing more accurate representations of microbial diversity, and identifying potentially novel species, MALDI-TOF’s ability to resolve taxonomic groups has other implications. *Bacillus cereus* is commonly found in the cleanroom environment, and is part of a taxonomic group that also includes *Bacillus thuringiensis* and the pathogenic *Bacillus anthracis.* These three species share >99% similarity between their 16S rRNA sequences (Yamada et al., 1999). The ability to rapidly and accurately identify isolates belonging to either of these species is not only beneficial to the medical community, but can result in a quicker response to a potential pathogen threat to engineers working in cleanroom environments.

### Future Applications

This custom MALDI-TOF MS database will be used for real-time, accurate, and high-throughput identification of bacterial isolates from spacecraft assembly cleanrooms. We will continue to update and curate this database as more unknown bacterial strains are isolated, which will increase the accuracy and spectrum of species we are able to identify using this technique (Rahi et al., 2016). The continual addition of type strains to the custom database will allow for increased confidence in identifying members of taxonomic groups. This custom database will aid in the detection of potentially novel species within our archived microbial collection. The rapid high throughput nature of MALDI-TOF MS will allow for the identification of potential contaminants and dangerous pathogens which will directly feed into better alerting engineers through the spacecraft assembly process. MALDI-TOF MS-based bacterial identification will be extremely cost-effective in performing high throughput screening of bacterial isolates for selection of unique bacterial strains for long-term preservation.

Though an open-access MALDI-TOF MS database, SpectraBank, is available, the submission of custom profiles is not possible (Bohme et al., 2012). We are willing to share our custom database upon request for research purposes, which will benefit other researchers working with similar microbial communities. Availability of a freely accessible online portal for MALDI-TOF MS database deposition and download will expand the use of MALDI-TOF MS for microbial ecology studies.

## Data Availability

16S rRNA sequences used in this study can be found on Genbank; accession numbers are provided in Supplementary Table S1. Raw MALDI-TOF MS spectral will be made available by the authors, without undue reservation, to any qualified researcher.

## Author Contributions

PV designed the project. HA and AS contributed equally to carrying out the research, performing the analysis, designing figures, and drafting the manuscript. JT, ML, and WS contributed to carrying out the research and analyzing the data. HA, AS, and PV wrote and critically reviewed the manuscript.

## Conflict of Interest Statement

The authors declare that the research was conducted in the absence of any commercial or financial relationships that could be construed as a potential conflict of interest.
