# Supplementary material for: Development of a Custom MALDI-TOF MS Database for Species-Level Identification of Bacterial Isolates Collected From Spacecraft and Associated Surfaces
Source: Front Microbiol. 2018 May 18;9:780. doi: 10.3389/fmicb.2018.00780 (PMC5968301; doi:10.3389/fmicb.2018.00780)
Supplement: Supplementary file 1 [file Table_1.DOCX]

**Supplementary Material**

S1. Strain names and NCBI accession numbers for 16S rRNA partial sequences from all 454 bacterial strains used in this study.

| Strain Name | NCBI Accession Number |
| --- | --- |
| SML_I15 | MG937540 |
| SML_I14 | MG937539 |
| SML_I17 | MG937541 |
| SML_I10 | MG937537 |
| SML_I13 | MG937538 |
| SML_I106 | MG937570 |
| SML_I102 | MG937568 |
| SML_I103 | MG937569 |
| SML_I112 | MG937571 |
| SML_I121 | MG937572 |
| SML_I123 | MG937573 |
| SML_I139 | MG937576 |
| SML_I138 | MG937575 |
| SML_I137 | MG937574 |
| SML_I140 | MG937577 |
| SML_I147 | MG937583 |
| SML_I145 | MG937581 |
| SML_I143 | MG937579 |
| SML_I146 | MG937582 |
| SML_I141 | MG937578 |
| SML_I144 | MG937580 |
| SML_I148 | MG937584 |
| SML_I152 | MG937585 |
| SML_I161 | MG937586 |
| SML_I163 | MG937587 |
| SML_I190 | MG937588 |
| SML_I25 | MG937545 |
| SML_I24 | MG937544 |
| SML_I22 | MG937543 |
| SML_I20 | MG937542 |
| SML_I27 | MG937547 |
| SML_I26 | MG937546 |
| SML_I29 | MG937548 |
| SML_I210 | MG937589 |
| SML_I218 | MG937590 |
| SML_I236 | MG937591 |
| SML_I248 | MG937592 |
| SML_I257 | MG937593 |
| SML_I260 | MG937594 |
| SML_I293 | MG937595 |
| SML_I32 | MG937550 |
| SML_I30 | MG937549 |
| SML_I35 | MG937551 |
| SML_I301 | MG937596 |
| SML_I311 | MG937597 |
| SML_I320 | MG937598 |
| SML_I327 | MG937600 |
| SML_I324 | MG937599 |
| SML_I345 | MG937601 |
| SML_I350 | MG937602 |
| SML_I56 | MG937557 |
| SML_I52 | MG937553 |
| SML_I51 | MG937552 |
| SML_I55 | MG937556 |
| SML_I53 | MG937554 |
| SML_I54 | MG937555 |
| SML_I60 | MG937558 |
| SML_I61 | MG937559 |
| SML_I75 | MG937564 |
| SML_I73 | MG937563 |
| SML_I72 | MG937562 |
| SML_I71 | MG937561 |
| SML_I70 | MG937560 |
| SML_I81 | MG937565 |
| SML_I90 | MG937566 |
| SML_I99 | MG937567 |
| MER TA 3-2 | KT719413.1 |
| MER TA 3B | KT719415.1 |
| MER TA 81 | KT719417.1 |
| MER_TA_13 | KT719422.1 |
| MER_TA_18 | KT719426.1 |
| MER TA 20 | KT719427.1 |
| MER_TA_21 | KT719428.1 |
| MER_TA_25 | KT719430.1 |
| MER TA 32A | KT719437.1 |
| MER TA 33.1 | KT719439.1 |
| MER TA 42 | KT719448.1 |
| MER TA 44 | KT719449.1 |
| MER TA 46 | KT719450.1 |
| MER TA 47.1 | KT719451.1 |
| MER TA 50 | KT719455.1 |
| MER TA 51 | KT719456.1 |
| MER TA 58 | KT719462.1 |
| MER_TA_60.1 | KT719463.1 |
| MER TA 61 | KT719466.1 |
| MER TA 66 | KT719470.1 |
| MER TA 69.1 | KT719473.1 |
| MER TA 70 | KT719475.1 |
| MER TA 71 | KT719476.1 |
| MER TA 78 | KT719484.1 |
| MER TA 79 | KT719485.1 |
| MER_TA_80.1 | KT719486.1 |
| MER TA 8-2 | KT719491.1 |
| MER TA 83 | KT719492.1 |
| MER TA 84 | KT719493.1 |
| MER TA 90 | KT719498.1 |
| MER_Ta_95 | KT719504.1 |
| MER TA 97 | KT719505.1 |
| MER TA 101 | KT719508.1 |
| MER TA 105 | KT719512.1 |
| MER TA 106 | KT719513.1 |
| MER TA 118 | KT719525.1 |
| MER_TA_131 | KT719534.1 |
| MER TA 132 | KT719535.1 |
| MER TA 136.1 | KT719539.1 |
| MER_TA_136.3.1 | KT719541.1 |
| MER TA 136-3-2 | KT719542.1 |
| MER TA 136-5 | KT719544.1 |
| MER_TA_137.3 | KT719547.1 |
| MER TA 137-4 | KT719548.1 |
| MER_TA_137.5 | KT719549.1 |
| MER_TA_138-1 | KT719550.1 |
| MER TA 143 | KT719556.1 |
| MER TA 144 | KT719557.1 |
| MER_TA_145 | KT719558.1 |
| MER TA 166 | KT719571.1 |
| MER_TA_172 | KT719576.1 |
| MER TA 175 | KT719579.1 |
| MER 1 | KT719585.1 |
| MER 2 | KT719586.1 |
| MER 10 | KT719594.1 |
| MER 12 | KT719596.1 |
| MER 19 | KT719600.1 |
| Mer 21 | KT719602.1 |
| MER 30 | KT719610.1 |
| MER 31 | KT719611.1 |
| MER 38 | KT719618.1 |
| MER_42 | KT719622.1 |
| MER 48 | KT719627.1 |
| MER 56 | KT719635.1 |
| MER 58 | KT719637.1 |
| MER 61 | KT719640.1 |
| MER 72 | KT719646.1 |
| MER 76.2 | KT719651.1 |
| MER_82 | KT719658.1 |
| MER_85 | KT719660.1 |
| MER_86 | KT719661.1 |
| MER 98 | KT719672.1 |
| MER 103 | KT719678.1 |
| MER 110 | KT719685.1 |
| MER 114.1 | KT719689.1 |
| MER 115 | KT719691.1 |
| MER 116 | KT719692.1 |
| MER 123C | KT719701.1 |
| MER 124b | KT719702.1 |
| MER 125C | KT719703.1 |
| MER 129 | KT719706.1 |
| MER 133 | KT719710.1 |
| MER 139 | KT719717.1 |
| MER 145 | KT719722.1 |
| MER_147A | KT719725.1 |
| MER_147B | KT719726.1 |
| MER_148A | KT719727.1 |
| MER 155 | KT719738.1 |
| MER 156 | KT719739.1 |
| MER 159 | KT719742.1 |
| MER 170 | KT719753.1 |
| MER_171 | KT719754.1 |
| MER 174B | KT719759.1 |
| MER 177 | KT719761.1 |
| MER 178 | KT719762.1 |
| MER 179 | KT719763.1 |
| MER 192a | KT719773.1 |
| MER 192b | KT719774.1 |
| MPF_14 | KT719788.1 |
| MPF 22 | KT719791.1 |
| MPF_56 | KT719798.1 |
| MPF 77 | KT719807.1 |
| MPF_80 | KT719808.1 |
| CF13.M1.1 | KT719816.1 |
| CF-18.M9 | KT719825.1 |
| CF-19.M7 | KT719826.1 |
| CFPSW.14.1 | KT719836.1 |
| CFPSW.15.2.2 | KT719841.1 |
| CFPSW15.2.3 | KT719842.1 |
| CFPSW.21.1 | KT719848.1 |
| CFPSW.22.1 | KT719849.1 |
| CFPSW.22.2 | KT719850.1 |
| MSL_3001 | KT719852.1 |
| MSL_3002 | KT719853.1 |
| MSL_3004 | KT719855.1 |
| MSL_3005 | KT719856.1 |
| MSL 3007 | KT719858.1 |
| MSL_3011 | KT719861.1 |
| MSL 3012 | KT719862.1 |
| MSL_3014 | KT719864.1 |
| MSL_3024 | KT719866.1 |
| MSL_3025 | KT719867.1 |
| MSL_3026 | KT719868.1 |
| MSL_3031 | KT719873.1 |
| MSL_3033 | KT719874.1 |
| MSL_3035 | KT719875.1 |
| MSL_3050 | KT719881.1 |
| MSL_3053 | KT719884.1 |
| MSL_3061 | KT719887.1 |
| MSL_3067 | KT719892.1 |
| MSL_3069 | KT719893.1 |
| MSL_3070 | KT719894.1 |
| MSL_3071 | KT719895.1 |
| AMY 13.1.2 | KT719901.1 |
| AMY_14.1.1 | KT719902.1 |
| AMY 14.1.2 | KT719903.1 |
| AMY 19.1.2 | KT719911.1 |
| AMY 2.1.1 | KT719912.1 |
| AMY 2.1.2.1 | KT719913.1 |
| AMY 20.1 | KT719916.1 |
| RA14A.10 | KT719947.1 |
| RA_14B.1 | KT719948.1 |
| Ph_02B5.1.2 | KT719963.1 |
| Ph_02B5.2 | KT719964.1 |
| Ph | KT719969.1 |
| Ph_03A6.1 | KT719974.1 |
| Ph_03A6.2.1 | KT719975.1 |
| Ph_04A4 | KT719980.1 |
| Ph_04A6.2 | KT719982.1 |
| Ph_04B12.2 | KT719984.1 |
| Ph04_B6.2 | KT719986.1 |
| Ph_15A | KT719988.1 |
| Ph_B9 | KT719996.1 |
| PHOENIX-FAIRING PF5-1.2 | KT719998.1 |
| PF5-1.3.1 | KT719999.1 |
| PF6-4.7 | KT720001.1 |
| PHOENIX-FAIRING PF9-10.1.2 DLE | KT720011.1 |
| PF4-9.1.3 | KT720018.1 |
| PHOENIX-FAIRING PF9-10.1.1 DLE | KT720019.1 |
| PF3-1.5 | KT720022.1 |
| PF6.4.3.1 | KT720023.1 |
| PF6-4.3.2 | KT720024.1 |
| PF2-12.2.3 | KT720030.1 |
| PF2_12.2.2 | KT720031.1 |
| Bacillus circulans_PF5 1.4 | KT720033.1 |
| PF2_12.2.1 | KT720034.1 |
| PF 1A.1.1.2 | KT720035.1 |
| PF24A_1.2 | KT720040.1 |
| PF2C_1 | KT720049.1 |
| PF3A.1 | KT720050.1 |
| PF3H_1.1 | KT720054.1 |
| PF3H_1.2.1 | KT720055.1 |
| PF3H_1.2.2 | KT720056.1 |
| PF 3H.2 | KT720057.1 |
| PF3L.1 | KT720061.1 |
| PF4A.1.1 | KT720063.1 |
| PF4A_1.2 | KT720064.1 |
| PF4A_3.1 | KT720067.1 |
| PF4C_1.1 | KT720068.1 |
| PF4i.1.3 | KT720085.1 |
| PF4i.2 | KT720086.1 |
| PF4J.1.3 | KT720089.1 |
| 10m_2.1 | KT720099.1 |
| Fairing 12L-1.1 | KT720102.1 |
| FAIRING 18B_1.1 | KT720104.1 |
| Fairing 19 B-1.1 | KT720107.1 |
| FAIRING 19B-1.2 DLE | KT720108.1 |
| FAIRING 1A-1 | KT720109.1 |
| FAIRING 2B-1.1 | KT720115.1 |
| 3B.1.1 | KT720125.1 |
| FAIRING 41A-3 DLE | KT720137.1 |
| FAIRING 4B-2.1 | KT720143.1 |
| 4G_1.1 | KT720145.1 |
| Fairing 7G-2.1 | KT720159.1 |
| 7G_3.1 | KT720160.1 |
| W12B_3.1 | KT720168.1 |
| W12B_4 | KT720169.1 |
| MECA_11-1.1 | KT720187.1 |
| MECA14-4.4 | KT720193.1 |
| M4.6 | KT720200.1 |
| M5.19 | KT720201.1 |
| M6-25 TEFLON | KT720204.1 |
| M10-2 | KT720207.1 |
| M33-22 | KT720210.1 |
| M34.11 | KT720211.1 |
| M36-32 | KT720212.1 |
| M38-4 | KT720214.1 |
| M38-12 | KT720215.1 |
| M38.20 | KT720217.1 |
| MM3.30f | KT720221.1 |
| T2.10 | KT720226.1 |
| T2-22D | KT720227.1 |
| T33-2 | KT720232.1 |
| T8.28 | KT720238.1 |
| T8-32 TEFLON RIBBON | KT720239.1 |
| T10-6 | KT720241.1 |
| Bacterium_T33.8 | KT720253.1 |
| T33.27a | KT720255.1 |
| T33.27b | KT720256.1 |
| V2.20 | KT720272.1 |
| V3-7 | KT720277.1 |
| V19-30 | KT720290.1 |
| V20-C2' | KT720291.1 |
| V39-9 | KT720297.1 |
| V39-23 | KT720300.1 |
| V39-29' | KT720301.1 |
| V-43_1a | KT720308.1 |
| V43-28 TEFLON RIBBON | KT720319.1 |
| V44_1'b | KT720321.1 |
| V44-8 | KT720324.1 |
| V44-9' | KT720327.1 |
| V47-11A | KT720342.1 |
| V47.11b | KT720343.1 |
| V47-23a | KT720344.1 |
| V48-13 | KT720347.1 |
| V48.17a | KT720348.1 |
| V48-26B | KT720352.1 |
| V50.13a | KT720353.1 |
| V50.13b | KT720354.1 |
| V51-25 | KT720356.1 |
| V57_11 | KT720361.1 |
| V59-32a | KT720368.1 |
| V61_2 | KT720373.1 |
| V61-7 TEFLON RIBBON | KT720374.1 |
| PF4C_1.1 | MG937603 |
| PF4i.1.3 | MG937605 |
| PF4i.2 | MG937604 |
| PF4J.1.3 | MG937607 |
| 10m_2.1 | MG937606 |
| Fairing 12L-1.1 | MG937608 |
| FAIRING 18B_1.1 | MG937664 |
| Fairing 19 B-1.1 | MG937665 |
| FAIRING 19B-1.2 DLE | MG937666 |
| FAIRING 1A-1 | MG937626 |
| FAIRING 2B-1.1 | MG937667 |
| 3B.1.1 | MG937668 |
| FAIRING 41A-3 DLE | MG937669 |
| FAIRING 4B-2.1 | MG937670 |
| 4G_1.1 | MG937671 |
| Fairing 7G-2.1 | MG937672 |
| 7G_3.1 | MG937673 |
| W12B_3.1 | MG937674 |
| W12B_4 | MG937675 |
| MECA_11-1.1 | MG937676 |
| MECA14-4.4 | MG937677 |
| M4.6 | MG937627 |
| M5.19 | MG937678 |
| M6-25 TEFLON | MG937679 |
| M10-2 | MG937680 |
| M33-22 | MG937681 |
| M34.11 | MG937682 |
| M36-32 | MG937683 |
| M38-4 | MG937684 |
| M38-12 | MG937685 |
| M38.20 | MG937686 |
| MM3.30f | MG937687 |
| T2.10 | MG937688 |
| T2-22D | MG937689 |
| T33-2 | MG937690 |
| T8.28 | MG937691 |
| T8-32 TEFLON RIBBON | MG937692 |
| T10-6 | MG937693 |
| Bacterium_T33.8 | MG937694 |
| T33.27a | MG937743 |
| T33.27b | MG937695 |
| V2.20 | MG937696 |
| V3-7 | MG937697 |
| V19-30 | MG937698 |
| V20-C2' | MG937699 |
| V39-9 | MG937700 |
| V39-23 | MG937701 |
| V39-29' | MG937702 |
| V-43_1a | MG937703 |
| V43-28 TEFLON RIBBON | MG937704 |
| V44_1'b | MG937705 |
| V44-8 | MG937706 |
| V44-9' | MG937707 |
| V47-11A | MG937708 |
| V47.11b | MG937709 |
| V47-23a | MG937710 |
| V48-13 | MG937610 |
| V48.17a | MG937612 |
| V48-26B | MG937611 |
| V50.13a | MG937609 |
| V50.13b | MG937711 |
| V51-25 | MG937712 |
| V57_11 | MG937713 |
| V59-32a | MG937714 |
| V61_2 | MG937715 |
| V61-7 TEFLON RIBBON | MG937716 |
| SML_M208 | MG937717 |
| SML_M209 | MG937718 |
| SML_M21 | MG937628 |
| SML_M210 | MG937719 |
| SML_M211 | MG937720 |
| SML_M213 | MG937721 |
| SML_M216 | MG937722 |
| SML_M217 | MG937723 |
| SML_M219 | MG937724 |
| SML_M22 | MG937629 |
| SML_M220 | MG937725 |
| SML_M223 | MG937726 |
| SML_M226 | MG937727 |
| SML_M229 | MG937728 |
| SML_M230 | MG937729 |
| SML_M231 | MG937730 |
| SML_M232 | MG937731 |
| SML_M234 | MG937732 |
| SML_M235 | MG937733 |
| SML_M236 | MG937734 |
| SML_M237 | MG937735 |
| SML_M240 | MG937736 |
| SML_M242 | MG937737 |
| SML_M246 | MG937738 |
| SML_M248 | MG937739 |
| SML_M250 | MG937740 |
| SML_M251 | MG937741 |
| SML_M252 | MG937742 |
| SML_M27 | MG937630 |
| SML_M37 | MG937613 |
| SML_M31 | MG937631 |
| SML_M40 | MG937614 |
| SML_M44 | MG937617 |
| SML_M43 | MG937616 |
| SML_M42 | MG937615 |
| SML_M4 | MG937623 |
| SML_M41 | MG937632 |
| SML_M45 | MG937633 |
| SML_M48 | MG937634 |
| SML_M49 | MG937635 |
| SML_M56 | MG937618 |
| SML_M5 | MG937624 |
| SML_M52 | MG937636 |
| SML_M54 | MG937637 |
| SML_M55 | MG937638 |
| SML_M59 | MG937639 |
| SML_M60 | MG937640 |
| SML_M61 | MG937641 |
| SML_M66 | MG937642 |
| SML_M67 | MG937643 |
| SML_M7 | MG937625 |
| SML_M74 | MG937619 |
| SML_M70 | MG937644 |
| SML_M72 | MG937645 |
| SML_M73 | MG937646 |
| SML_M75 | MG937647 |
| SML_M76 | MG937648 |
| SML_M77 | MG937649 |
| SML_M79 | MG937650 |
| SML_M88 | MG937621 |
| SML_M87 | MG937620 |
| SML_M80 | MG937651 |
| SML_M81 | MG937652 |
| SML_M84 | MG937653 |
| SML_M85 | MG937654 |
| SML_M86 | MG937655 |
| SML_M89 | MG937656 |
| SML_M92 | MG937657 |
| SML_M94 | MG937658 |
| SML_M95 | MG937659 |
| SML_M96 | MG937660 |
| SML_M97 | MG937661 |
| SML_M98 | MG937662 |
| SML_M99 | MG937663 |
| SML_M1 | MG937622 |
| SML_M2 | MG937744 |
| MER 138 | KT719716.1 |
| V44-14''' TEFLON RIBBON | KT720319.1 |
